# Individual encounter data of six African carnivore species optimized for multi-species density estimation

**DOI:** 10.1038/s41597-026-07332-x

**Published:** 2026-06-22

**Authors:** Laura C. Gigliotti, Gates Dupont, Chris Sutherland, Charli Pretorius, Simon Naylor, Kerrin Allan, Gonçalo Curveira-Santos

**Affiliations:** 1 U.S. Geological Survey, West Virginia Cooperative Fish and Wildlife Research Unit, West Virginia University, Morgantown, WV USA; 2 Department of Ecology & Evolutionary Biology, Princeton University, Princeton, NJ USA; 3 Centre for Research into Ecological and Environmental Modelling, University of St Andrews, St Andrews, UK; 4 andBeyond Phinda Private Game Reserve and Munywana Conservancy, Hluhluwe, South Africa; 5 CIBIO Research Center in Biodiversity and Genetic Resources, InBIO Associated Laboratory, Universidade do Porto, Vairão, Portugal; 6 BIOPOLIS Program in Genomics, Biodiversity and Land Planning, CIBIO, Vairão, Portugal

**Keywords:** Community ecology, Biodiversity, Conservation biology

## Abstract

The ability to estimate abundances of multiple wildlife species within an area is valuable for both conservation and ecological inquiry. Spatially explicit capture–recapture (SCR) methods are commonly used to obtain reliable population size estimates, particularly for low-density and individually identifiable carnivore species. However, estimating abundance within multi-species communities poses a methodological challenge as survey designs and analytical tools are primarily tailored for single target species. Here, we present a dataset of spatially referenced individual encounter histories of six carnivore species with varying space requirements (lion, *Panthera leo*; leopard, *Panthera pardus*; spotted hyena, *Crocuta crocuta*; cheetah, *Acinonyx jubatus*; serval, *Leptailurus serval*; large-spotted genet, *Genetta tigrina*). These data were collected in a South African game reserve using a camera trap array optimized for multi-species density estimation using SCR methods. This dataset will be a valuable resource for studying spatial processes among potentially interacting carnivores without the common pitfalls that come with by-catch data of non-target species, and will provide a much-needed case study for the further development of multi-species statistical method development.

## Background & Summary

Wildlife conservation is increasingly embracing community-level and network-based approaches over single-species management strategies^1^. Passive wildlife sampling methods, particularly camera-trapping, are capable of collecting concurrent data on multiple species within a community. While these community-level monitoring approaches often provide valuable insights into species richness, distribution, or relative abundances, they typically fall short in delivering the data required to produce estimates of absolute abundance and demographic parameters (e.g., survival, recruitment) across multiple species^2^. For species with individual markings, camera data analyzed within a Spatial Capture-Recapture (SCR) framework has emerged as a key tool to generate reliable estimates of these parameters^3,4^. Yet, despite its efficacy, SCR is still seldom applied within multi-species frameworks^5^, often due to survey design considerations.

Density estimation using SCR relies on spatially referenced detections of individual animals^3,4^. Critically, SCR requires that at least some individuals are detected at multiple locations, and therefore that the array of detectors is often deployed with spacing proportional to the space use of the focal species^6,7^. However, when trying to monitor multiple species simultaneously, in cases where the body size, home range size, and space-use of the assemblage of species varies markedly, clearly what is optimal for one species will be sub-optimal for another^6,8^. Although recent analytical approaches such as Random Encounter Models (REM), Camera Trap Distance Sampling, and time- and space-to-event models have been developed to estimate the densities of multiple species from camera trap data^9–11^, these approaches can be limited in their application to low-density or difficult-to-detect species^12,13^ and their general performance is mixed.

We previously developed an optimization procedure for SCR study design that considers the ecological characteristics of a suite of focal species and generates data that yields robust estimates of density for each^7^. Using our optimized camera trap survey design, we conducted a camera trap study to collect data on six individually-identifiable carnivore species (lion, *Panthera leo*; leopard, *Panthera pardus*; spotted hyena, *Crocuta crocuta*; cheetah, *Acinonyx jubatus*; serval, *Leptailurus serval*; large-spotted genet, *Genetta tigrina*) in a game reserve in South Africa. We identified individuals for each species to generate spatially explicit encounter histories that can be analyzed using SCR.

**Fig. 1:**
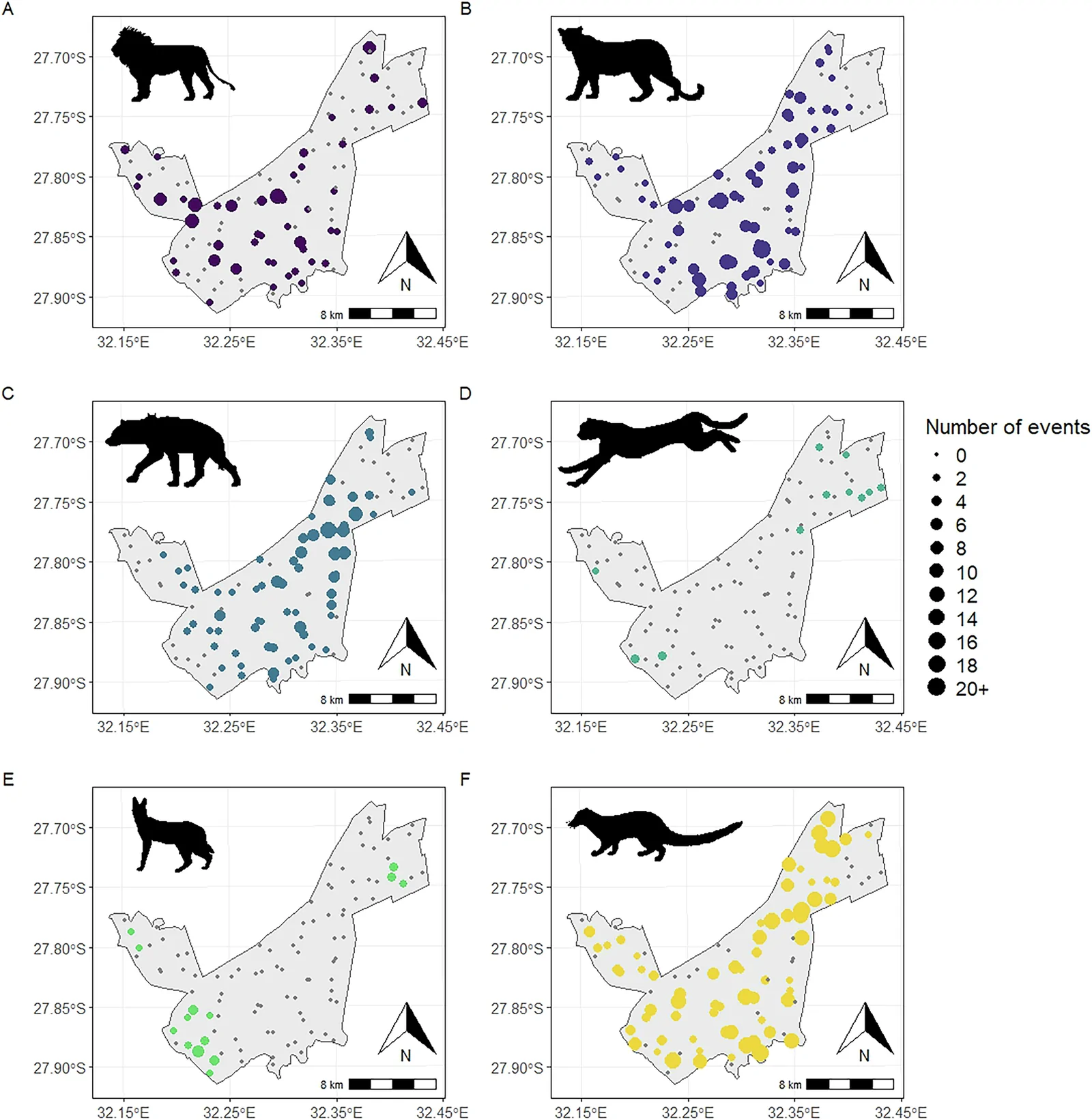
Spatial distribution of independent photo events of individually-identifiable (**A**) lions (*Panthera leo*), (**B**) leopards (*Panthera pardus*) (**C**) spotted hyenas (*Crocuta crocuta*), (**D**) cheetahs (*Acinonyx jubatus*), (**E**) servals (*Leptailurus serval*), and (**F**) large-spotted genets (*Genetta tigrina*), Munywana Conservancy, KwaZulu-Natal, South Africa, 2021–2022. Gray dots indicate sites where a species was not detected.

To date, there are no publicly available camera trap datasets on multiple species collected simultaneously that i) are specifically optimized for analysis using SCR and ii) meet modelling assumptions for all species. Our approach satisfies both of these conditions and thus provides a valuable resource for studying spatial processes in potentially interacting carnivores without the common pitfalls that come with by-catch data. Additionally, our work contributes empirical data towards the enduring challenge of designing multi-species monitoring designs and offers a unique real-world case study for evaluating future multi-species method developments. The dataset we present here includes the detection histories of 438 individual animals across six carnivore species (Fig. 1, Table 1), and camera trap deployment information. With these data, researchers can investigate a diversity of questions ranging from quantitative developments in multi-species density estimation, to ecological investigations into carnivore community structure and species co-occurrence.

**Table 1:** Descriptive statistics of the multi-species detection history dataset, Munywana Conservancy, KwaZulu-Natal, South Africa, 2021–2022.

Species	Total identifiable detections	Number of individuals	Mean detections per individual (range)	Mean sites per individual (range)
Lion (*Panthera leo*)	141	21	6.71 (2–23)	4.52 (1–13)
Leopard (*Panthera pardus*)	254	48	5.29 (1–21)	2.77 (1–11)
Spotted hyena (*Crocuta crocuta*)	195	51	3.82 (1–12)	3.43 (1–9)
Cheetah (*Acinonyx jubatus*)	20	6	3.33 (1–7)	3.00 (1–6)
Serval (*Leptailurus serval*)	33	11	3.00 (1–9)	2.55 (1–8)
Large-spotted genet (*Genetta tigrina*)	614	301	2.04 (1–17)	1.07 (1–3)

## Methods

### Study area

We collected data in the Munywana Conservancy in KwaZulu-Natal, South Africa (−27.82112°, 32.30789°). The Conservancy is 285 km^2^ and is surrounded by electrified game fencing. The dominant habitat type is broad-leaf woodlands, with open grasslands and semi-open wooded-grasslands interspersed throughout the Conservancy. Elevation in the Conservancy ranges from 4–201 m above sea level. The Conservancy exists in a subtropical climate with warm, dry winters (April – September), and hot summers (October – March) during which time the majority of the rain falls. The Conservancy is managed as an ecotourism reserve, with several individual private properties that are managed as a single contiguous area without internal fences. Hunting is not allowed on the Conservancy.

### Camera-trapping array

Locations of camera trapping sites were determined using a 2-stage multi-species optimization, as described in Curveira-Santos *et al*.^7^, but we provide a brief summary here. We created our design using a two-stage allocation of 100 monitoring sites. First, 60 sites were allocated based on optimal placement for leopards (selected for their large space use requirements and the motivation for estimating leopard densities within the focal reserve^14^) using a space-filling criterion^6^, a baseline detection probability (p_0_) of 0.2 and a spatial scale parameter (σ) of 1200 m. Second, we treated the locations generated by the ‘leopard allocation’ (step 1) as fixed points, and allocated the remaining 40 sites to target carnivores with smaller space requirements (i.e. servals and large-spotted genets). This second optimization was based on a space-constraining criterion^6^, a baseline detection probability (p_0_) of 0.2 and a spatial scale parameter (σ) of 500 m. Our design optimization was created using the *scrdesignGA* function in the *oSCR* R package^15^. When the two stages of our optimization were combined (i.e. considering all 100 sites), the mean distance to the nearest camera site was 877 m (range = 218 m–1753 m).

### Data collection

We placed unbaited, paired white-flash camera traps (Cuddeback Professional Color model 1347 or Spartan Lumen, with both cameras in a pair being of the same brand) at our 100 sites. At each site, we placed one camera on each side of a road, such each camera was angled 60–70° from the road, and staggered approximately 2 m from the opposite camera^14^. We set cameras 30 cm high on stakes or trees that were 2-3 m from the target animal passage zone. Cameras at each site were active for a minimum of 45 days and we used a rotational deployment, such that approximately half the sites were active at a time^16^. Our survey ran from 17 September 2021 – 11 January 2022, with sites in the southern part of the survey (n = 54 sites) active from 17 September 2021 – 8 November 2021, and sites in the northern part of the survey (n = 46 sites) active from 12 November 2021 – 11 January 2022.

### Data processing

We identified species present in all photographs using TrapTagger (WildEye, traptagger.co.uk), and identified individuals within each species using African Carnivore Wildbook^17^, which uses the HotSpotter algorithm to match individual animals based on spot patterns^18^. We manually vetted all matches identified by the program. Because lions do not have individually identifiable spot patterns, we identified individuals using whisker spots and scars. For lions, leopards, and cheetahs we also recorded the sex of the individual based on the visible external genitalia and secondary sexual traits (e.g., male’s mane or dewlap). Our dataset only includes data from adult animals; we excluded juveniles based on size and being photographed with an adult.

## Data Record

All data are publicly available in an Open Science Framework repository^19^. These data include:

Individual Identification Database (MultiSpecies_Individual_ID.csv; 1257 data rows):

- Individual_ID: Text. Individual animal ID associated with each specific capture at a site
- Common_Name: Text. Common name of the species.
- Scientific_Name: Text. Scientific name of the species
- Site: Text. Site name that the individual was recorded by a camera trap. Site names range from MYW01 to MYW100 and match up with the Site names in the Camera Operation Database
- Date: Camera trap capture date. dd/mm/yyyy format.
- Sex: Text. Sex of the individual. Sex determination from camera trap photos is only possible for lions, leopards, and cheetahs. Options are F (female), M (male), and U (unknown). Sex determination from camera trap photos is not possible for spotted hyenas, servals, and genets, so they are all labeled as U (unknown).
- Side: Text. Label indicating if both sides of an individual are known (“both”), or only the right (“right”) or left (“left”) sides.

Camera Operation Database (CamTrap_Operation_Final.csv; 100 data rows):

- Site: Site name stored as text. Site names range from MYW01 to MYW100
- UTM_x: Numeric x-coordinate of the spatial location of the camera trap site recorded in UTM Zone 36S
- UTM_y: Numeric y-coordinate of the spatial location of the camera trap site recorded in UTM Zone 36S
- Half: Text. Used to label which half of the survey a camera site was associated with. Options include “North” (active from 12 November 2021 – 11 January 2022) and “South” (active from 17 September 2021 – 8 November 2021).
- Brand: Text. Camera trap brand used for each specific site. Options include “Cuddeback” (indicating Cuddeback Professional Color model 1347) and “Spartan” (indicating Spartan Lumen)
- Grid_Allocation: Text. Label indicating if the site was generated as part of the stage-1 optimization (“Leopard”) or the stage-2 optimization (“Small”).
- 9/17/2021 – 1/11/2022: Individual columns indicating camera trap daily operation status. Options include 1 (at least 1 camera trap was deployed and operational at a site), 0 (at least 1 camera trap was deployed at a site, but no cameras were operational), and NA (no camera traps were deployed at a site).

Study Area Shapefile

- The shapefile Full_Phinda_2018_UTM.shp (Coordinate Reference System: WGS 1984 UTM Zone 36S), is the boundary of the reserve.

## Technical Validation

We manually verified the species identified by TrapTagger and removed any photos that we were not able to identify with certainty. Individual identification was performed by two individuals (L.C.G and C.P.) with many years of experience in identifying carnivores from spot patterns. We only included data from individuals identified with consensus of the two reviewers. We were able to assign an identity to the majority of events of lions (88.7%), leopards (86.7%), spotted hyenas (67.9%), cheetahs (100%), servals (89.2%), and large-spotted genets (67.1%)^7^. Lions and cheetahs have been extensively monitored in the Conservancy since the early 1990’s, and the Conservancy maintains an ID-kit of all lions and cheetahs within the Conservancy. Thus, we were able to use the ID-kit to further verify our lion and cheetah individual identifications.

## Usage Notes

### Spatially-explicit capture recapture

These data are formatted to be able to conduct SCR analyses to estimate the density of multiple species^4^. To be able to conduct this type of analysis, a few considerations need to be taken. First, the fence in this system is permeable for leopards, spotted hyenas, servals, and genets, but not permeable for lions and cheetahs^20,21^; thus the state space needs to be restricted to only within the Conservancy boundary for the latter group of species. Second, for all species, we include detection/non-detection at the individual level, but we do not provide the number of individuals in a single capture event (e.g. multiple lions could be photographed together). Third, we did not include sex or partial identities (i.e. if just one side was known for an individual) into our models, but future research could explore these extensions with the information included in our data.

### Other analyses

We expect that these data could be used for analyses other than density estimation using SCR models; for instance, to test some ‘unmarked’ abundance models or common abundance proxies (e.g., occupancy) against SCR estimates. However, if other analyses are used, it is important to take into account potential biases in the data, as well as supplemental data that might be required. For example, some unmarked models require information on species’ movement rates, which would necessitate using values in the literature to parameterize the model^11,22^. Similarly, we did not measure the specific camera viewshed for each camera site, but coarse-scale viewshed metrics could be derived from camera brand specifications^23^. Based on logistical considerations, cameras were placed on roads. Although this camera placement was suitable for most of our target carnivore species, which are known to have high detection rates on roads^24^, it interferes with a common model assumption of random camera placement relative to animal movement^11^. Camera placement on roads might have also contributed to reduced sample sizes for species such as cheetahs that tend to use roads less frequently^25^. The non-random placement would violate assumptions of some unmarked density models, but could be accounted for analytically^26^. Additionally, the non-random and clustered distribution of individual camera traps promoted by our optimized design for multi-species density estimation using SCR models, might be unsuitable for other types of analyses. For example, many cameras are located closer together than is recommended for occupancy studies^27^ or random encounter models^28^, thus requiring analytical methods to account for this spatial autocorrelation^26^, such as spatially-explicit occupancy models^29^.

## Data Availability

All data described here are publicly available in an Open Science Framework repository: Individual spatial encounter data of six carnivore species optimized for multi-species density estimation (https://doi.org/10.17605/OSF.IO/4GRE3)^19^.

## Code availability

The code used to process data to create individual capture histories and run species-specific SCR models are available in an Open Science Framework repository^30^ and additional details about running SCR models using these data can be found in Curveira-Santos *et al*.^7^.
